# Estrogen receptor β ligation inhibits Hodgkin lymphoma growth by inducing autophagy

**DOI:** 10.18632/oncotarget.14338

**Published:** 2016-12-28

**Authors:** Marina Pierdominici, Angela Maselli, Silvia L. Locatelli, Laura Ciarlo, Giuseppa Careddu, Mario Patrizio, Barbara Ascione, Antonella Tinari, Carmelo Carlo-Stella, Walter Malorni, Paola Matarrese, Elena Ortona

**Affiliations:** ^1^ Department of Cell Biology and Neurosciences, Istituto Superiore di Sanità, Rome, Italy; ^2^ Department of Therapeutic Research and Medicine Evaluation, Istituto Superiore di Sanità, Rome, Italy; ^3^ Department of Oncology and Hematology, Humanitas Cancer Center - Humanitas Clinical and Research Center, Milano, Italy; ^4^ Department of Technology and Health, Istituto Superiore di Sanità, Rome, Italy

**Keywords:** Hodgkin lymphoma, estrogen receptor β, autophagy, DRAM2, tumor growth

## Abstract

Although Hodgkin lymphoma (HL) is curable with current therapy, at least 20% of patients relapse or fail to make complete remission. In addition, patients who achieve long-term disease-free survival frequently undergo infertility, secondary malignancies, and cardiac failure, which are related to chemotherapeutic agents and radiation therapies. Hence, new therapeutic strategies able to counteract the HL disease in this important patient population are still a matter of study. Estrogens, in particular 17β-estradiol (E2), have been suggested to play a role in lymphoma cell homeostasis by estrogen receptors (ER) β activation. On these bases, we investigated whether the ligation of ERβ by a selective agonist, the 2,3-bis(4-hydroxyphenyl)-propionitrile (DPN), could impact HL tumor growth. We found that DPN-mediated ERβ activation led to a reduction of *in vitro* cell proliferation and cell cycle progression by inducing autophagy. In nonobese diabetic/severe combined immunodeficient (NOD/SCID) mice engrafted with HL cells, ERβ activation by DPN was able to reduce lymphoma growth up to 60% and this associated with the induction of tumor cell autophagy. Molecular characterization of ERβ-induced autophagy revealed an overexpression of damage-regulated autophagy modulator 2 (DRAM2) molecule, whose role in autophagy modulation is still debated. After ERβ activation, both DRAM2 and protein 1 light chain 3 (LC3), a key actor in the autophagosome formation, strictly interacted each other and localized at mitochondrial level.

Altogether these results suggest that targeting ERβ with selective agonists might affect HL cell proliferation and tumor growth via a mechanism that brings into play DRAM2-dependent autophagic cascade.

## INTRODUCTION

Hodgkin lymphoma (HL) is a B-cell malignancy whose etiology still remains poorly understood [1]. Both progression-free survival and overall survival of patients with HL have strongly been improved in the last decades by the combination of chemotherapy and irradiation. However, about 20% of patients relapse or fail to undergo complete remission and their prognosis is quite poor [2]. Furthermore, chemotherapy and irradiation may affect fertility and cause cardiac failure and secondary malignancies, such as breast cancer [3–5]. Therefore, novel therapeutic approaches improving clinical outcome with limited adverse effects are needed.

Previous evidence supports an anti-proliferative role of estrogen, in particular 17β-estradiol (E2), in lymphomas [6]. The biological effects of E2 are mediated by two specific intracellular receptors, estrogen receptor (ER)α and ERβ, which function as ligand-activated nuclear transcription factors producing genomic effects [7]. Activation of ERα exerts pro-proliferative effects, whereas ERβ activates an anti-proliferative signaling pathway through the execution of a specific and complex gene expression program [8, 9]. ERβ represents the main ER expressed in lymphoid malignancies while ERα levels are very low or undetectable [6, 10–12]. ERβ activation by selective agonists was found to inhibit non Hodgkin lymphoma (NHL) growth, both in cell-based- and mouse-models, mainly reducing cell proliferation [10, 12]. Furthermore, ERβ agonists were able to inhibit lymphoma vascularization and dissemination in mice [12] further supporting their potential use as therapeutic tools. However, the precise molecular mechanisms of ERβ-activated pathways underlying these effects still remain unidentified. Interestingly, recent studies suggest that ERβ ligation could also trigger the lysosome-mediated pathway named autophagy [13, 14]. This is a selective catabolic process involved both in basal turnover of altered cellular components and in counteracting stressful dysmetabolic conditions. The Janus-faced influence of autophagy on the initiation and progression of cancers has still to be well established. It has in fact been suggested to exert anti- or pro-tumor functions in a context-dependent way [15, 16]. Interestingly, defective autophagy pathways have been implicated in lymphoid malignancies and autophagic machinery-targeted agents, acting as inducers of autophagy, are emerging as promising agents for the treatment of lymphomas (for a review see [17]). However, no data have directly connected the anti-proliferative activity of ERβ in lymphoma to the modulation of autophagy.

Up to date, the role of ERβ activation in HL is poorly understood. Therefore, in the present work, we first demonstrated the ability of ERβ ligation to exert an anti-proliferative effect both *in vitro*, in a panel of HL cells, and *in vivo*, in nonobese diabetic/severe combined immunodeficient (NOD/SCID) mice grafted with HL cells. Then we revealed the role of ERβ ligation to inhibit HL growth by inducing autophagy, analyzing in some detail the molecular mechanisms underlying this effect.

## RESULTS

### ERβ selective agonist DPN blocks proliferation and cell cycle progression in HL cells

**Figure 1:**
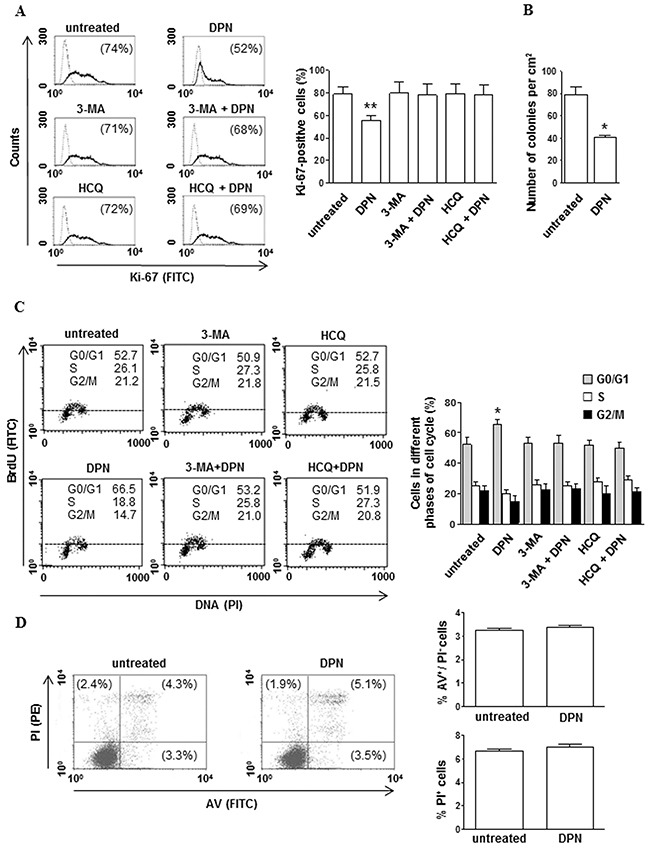
DPN reduces cell proliferation and alters cell cycle progression in HL cells **A**. Cell proliferation was evaluated by flow cytometry measuring Ki-67 nuclear antigen expression in L-428 cells treated or not with 10 nM DPN for 48 hours in the presence or absence of 3-MA or HCQ. Results from one representative experiment out of 3 are shown (left). Data are also reported as mean ± SD (right), **, *p* < 0.01 *versus* untreated cells. **B**. The effects of DPN (10 nM) on L-428 long-term survival was determined by *in vitro* colony formation assay. The mean (± SD) values correspond to three independent experiments, *, *p* < 0.05. **C**. Cell cycle progression was evaluated by flow cytometry using the BrdU/anti-BrdU method in synchronized L-428 cells treated or not with 10 nM DPN for 48 hours in the presence or absence of 3-MA or HCQ. Results from one representative experiment out of 3 are shown (left). Data are also reported as mean ± SD (right), *, *p* < 0.05 *versus* untreated cells. **D**. Apoptosis/necrosis assay involving dual staining with AV and PI was carried out using flow cytometry in L-428 cells treated or not with 10 nM DPN for 48 hours. Results from one representative experiment out of 3 are shown (left). Numbers reported represent the percentages of AV positive/PI negative (early apoptotic, bottom right quadrant) and PI positive (late apoptotic or necrotic cells, top right and left quadrants). Data are also reported as mean ± SD (right).

To elucidate the role of ERβ ligation in HL growth, we first investigated whether ERβ activation by the agonist 2,3-bis(4-hydroxyphenyl)-propionitrile (DPN) could affect cell proliferation and cycle progression in human HL cell lines, i.e., B-cell HL L-428 and KM-H2 and T-cell HL L-540 and HDLM-2. Interestingly, after treatment of HL cells with DPN we observed a significant decrease of the percentage of Ki-67-positive cells (L-428 cells, Figure 1A, *p* < 0.01 *versus* untreated cells; KM-H2, L-540 and HDLM-2 cells, Supplementary Figure 1A, *p* < 0.05 *versus* untreated cells). Additionally, we investigated the effect of DPN on long-term survival of L-428 cells (Figure 1B). A ten-day exposure to DPN reduced the number of L-428 colonies down to 50% compared to untreated cells (Figure 1B). An increase of the percentage of cells in G0/G1 phase, indicating a sharp slowdown in the cell cycle progression, was also observed (L-428 cells, *p* < 0.05 *versus* untreated cells, Figure 1C; KM-H2, L-540 and HDLM-2 cells, Supplementary Figure 1B, *p* < 0.05 *versus* untreated cells). No differences were observed in terms of early apoptotic [annexin V (AV) positive/propidium iodide (PI) negative] and late apoptotic or necrotic cells (PI positive) in HL cells treated or not with DPN (L-428 cells, Figure 1D; KM-H2, L-540 and HDLM-2 cells, data not shown).

### ERβ selective agonist DPN induces autophagy in human HL cells

**Figure 2:**
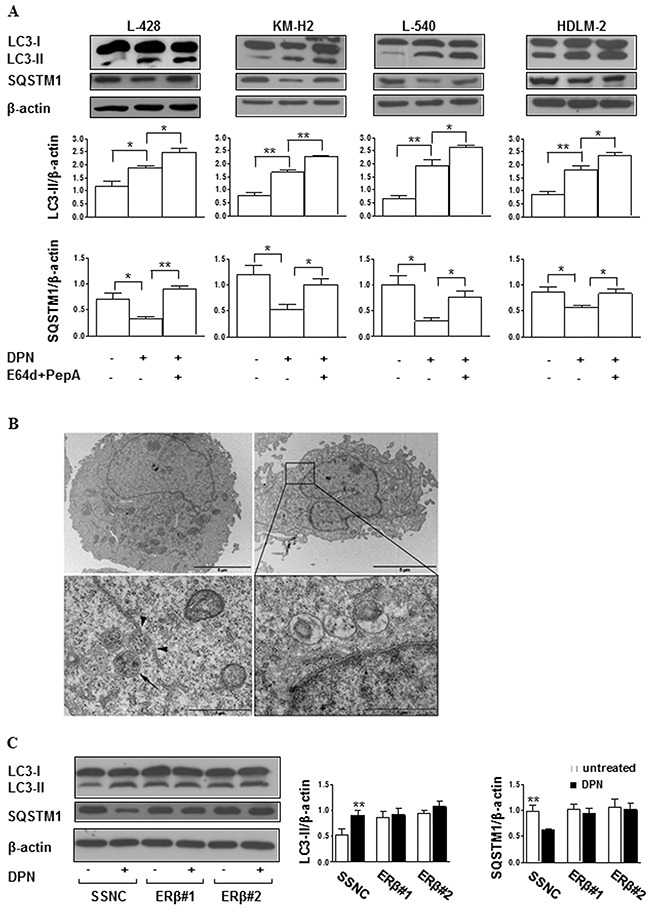
DPN induces autophagy in HL cells **A**. Western blot analysis of the autophagic markers LC3-II and SQSTM1 in cell lysates from L-428, KM-H2, L-540, and HDLM-2 cells treated or not with 10 nM DPN for 24 hours, in the presence or absence of the lysosomal inhibitors E64d and PepA. For each cell line, blots shown are representative of 3 independent experiments (top). Densitometry analysis of specific protein levels relative to β-actin is also shown (bottom). Values are expressed as mean ± SD; *, *p* < 0.05 and **, *p* < 0.01 *versus* untreated cells. **B**. TEM analysis performed on L-428 cells treated or not for 24 hours with 10 nM DPN and observed by a Philips 208 electron microscope at 80 kV, equipped with Mega Wiew II, Olympus soft imaging solutions. The images were acquired by the specific software ITEM tem imaging platform. In the top panels (at magnification of 6300x) untreated (left) and DPN-treated (right) cells are shown. When observed at a higher magnification (32000x, bottom panels), DPN-treated cells displayed several autophagic vacuoles (left) as well as autophagolysosomes (right). To note, autophagic vacuoles showing the characteristic double membrane (arrow) appeared to be close to endoplasmic reticulum (arrowheads, left). Results from one representative experiment out of 3 are shown. **C**. Western blot analysis of LC3-II levels in cell lysates from L-428 cells transfected with Silencer Select Negative Control (SSNC) or ERβ siRNA (ERβ#1 and ERβ#2) and treated or not with 10 nM DPN for 24 hours. Blots shown are representative of 3 independent experiments (left). Densitometry analysis of LC3-II levels relative to β-actin is also shown (right). Values are expressed as mean ± SD. **, *p* < 0.01 *versus* untreated cells.

Because ERβ ligation was reported to induce autophagy in different transformed human cells [13, 14], we evaluated whether DPN could modulate autophagy in HL cells. To this aim, we analyzed the expression level of the autophagic markers microtubule-associated protein 1 light chain 3 (LC3)-II and (sequestosome 1) SQSTM1 by western blot. LC3 is an essential factor for autophagosome formation, its unlipidated cytosolic form is called LC3-I, whereas the lipidated form is referred as to LC3-II and localizes to autophagosomal membranes throughout the maturation process of the autophagosome. For this reason, the increase of LC3-II is commonly used for monitoring autophagy levels, together with the decrease of SQSTM1, an ubiquitin-binding protein forming protein aggregates degraded by autophagy [18]. We found that DPN was able to significantly increase LC3-II expression levels in all tested HL cells (Figure 2A). A concomitant SQSTM1 decrease was observed (Figure 2A) suggesting the induction of autophagy. These results also suggested that ERβ-dependent autophagy induction was a typical behavior of HL tumor cells not depending to their B or T cell origin. In order to gain further insights into the mechanism of DPN-induced autophagy, a LC3 turnover assay, employing the lysosomal inhibitor E64d and pepstatin A (PepA) co-treatment, was performed (Figure 2A). In fact, it is well known that LC3-II can accumulate because of increased upstream autophagosome formation or impaired downstream autophagosome lysosome fusion [18]. To distinguish between these two possibilities, we assayed DPN-induced LC3-II accumulation in the presence or absence of the above mentioned lysosomal protease inhibitors. When DPN treatment occurred in the presence of E64d and PepA, DPN-induced upregulation of LC3-II levels was potentiated, this being consistent with an increased upstream autophagosome formation.

**Figure 3:**
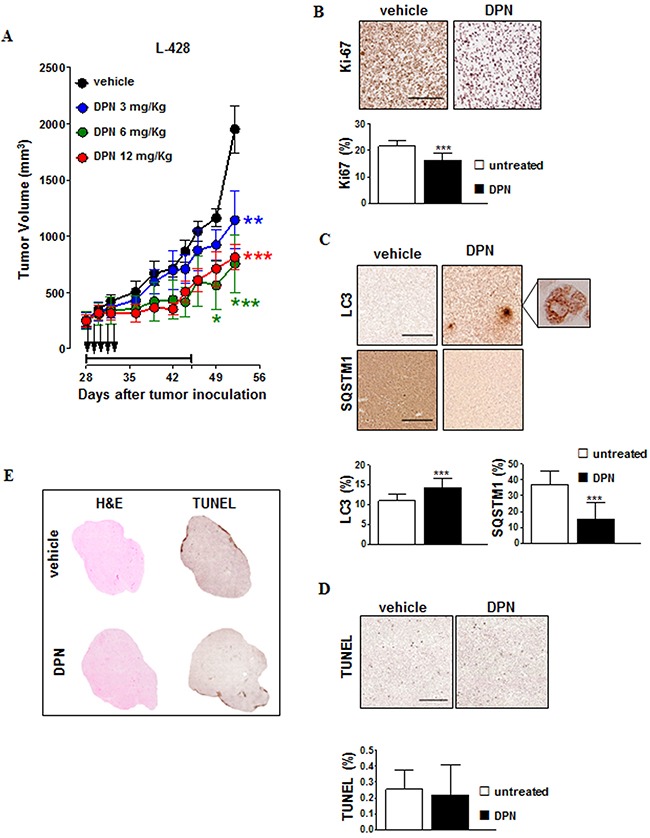
DPN induces growth inhibition and autophagy in HL xenografts in NOD/SCID mice **A**. NOD/SCID mice bearing 100-mm^3^ L-428 tumor nodules were randomly assigned to receive 15-day treatment with DPN by intraperitoneal injection 5 days/week for 3 weeks (3 mg/kg, blue; 6 mg/kg, green; 12 mg/kg, red) or vehicle control (black). Black arrows indicate DPN treatment administration, whereas treatment duration is indicated by horizontal capped black line (days 28-46). The mean (± SEM) tumor volumes are shown. *, *p* < 0.05; **, *p* < 0.001; ***, *p* < 0.0001 compared with the vehicle controls. **B**. Ki-67 and **C**. LC3 and SQSTM1 staining of L-428 tumors treated with DPN (12 mg/kg/day, 5 days) or vehicle control. In Ki-67-, LC3-, and SQSTM1-stained sections brown staining represents a positive signal within the tumor (top). Objective lens, 0.75 numerical aperture (NA) dry objective; original magnification, 20x. Scale bar, 100 µm. Enlarged picture (500x) shows localized distinct puncta (C). For each parameter, data are also represented as mean ± SD (bottom). Results are calculated as mean from 15 microscope fields for each sample, 3 mice per group. Images were analyzed using Image-Pro Analyzer software. Original magnification 20X. Statistical analysis: *, *p* < 0.001. **D**. In L-428 tumors treated with DPN (12 mg/kg/day, 5 days) or vehicle control, apoptotic tumor cells were detected by TUNEL staining and were revealed as brown nuclei using 3,3’-diaminobenzidine for light microscopy analysis (top). Objective lens, 0.08 NA dry objective; original magnification, 20x. Scale bar, 50 µm. Digitally acquired TUNEL stained sections were analyzed using ImageJ for quantification of the apoptotic percentage (mean ± SD, bottom). At least three sections from different animals were analyzed. **E**. Representative histological images of entire tumor sections from L-428 tumors treated with DPN (12 mg/kg/day, 5 days) or vehicle control. Tumor tissue morphology was detected via hematoxylin and eosin (H&E) staining. The tumor necrotic areas were detected via TUNEL staining and were visualized as brown. Objective lens, 0.08 NA dry objective; original magnification, 2x.

To further confirm autophagy induction, ultrastructural analyses were carried out by transmission electron microscopy (TEM) in L-428 and L-540 cells, chosen as representative of HL of B and T cell-origin, respectively. The presence of autophagic vacuoles containing structures that can clearly be identified as cytoplasmic, such as partially degraded endoplasmic reticulum and cell debris, was observed in DPN-treated L-428 (Figure 2B) and L-540 (Supplementary Figure 2) cells. The specificity of DPN binding to ERβ and its role in autophagy induction was confirmed by transfection of L-428 and L-540 cells with ERβ small interfering RNA (siRNA). Twenty-four hours after siRNA addition, ERβ expression decreased by about 50% as assessed by western blot (Supplementary Figure 3A and Figure 3B for L-428 and L-540 cells, respectively). Notably, no significant changes of LC3-II and SQSTM1 levels were found after DPN treatment in ERβ-silenced cells (L-428, Figure 2C; L-540 cells, Supplementary Figure 3C). Altogether, the current set of experiments demonstrated that ERβ activation leads to autophagy induction in HL cells interfering with autophagosome formation.

### Autophagy inhibition impairs the effects of ERβ selective agonist DPN on proliferation and cell cycle progression in human HL cells

To determine whether autophagy mediates DPN-dependent inhibition of proliferation and cell cycle progression, before DPN administration HL cells were pretreated with i) 3-methyladenine (3-MA), known to inhibit early phases of autophagy by blocking autophagosome formation *via* the inhibition of type III phosphatidylinositol 3-kinases, or ii) hydroxychloroquine (HCQ), a specific inhibitor of the late phases of autophagy as it raises the lysosomal pH, which leads to inhibition of both fusion of autophagosome with lysosome and lysosomal protein degradation. We found that both 3-MA and HCQ i) effectively inhibited autophagy (data not shown), ii) did not alter *per se* proliferation and cell cycle progression, but iii) were able to hinder the effects of DPN on proliferation and cell cycle progression (L-428, Figure 1A and Figure 1B; KM-H2, L-540 and HDLM-2 cells, Supplementary Figure 1A and Figure 1B).

### ERβ selective agonist DPN inhibits human lymphoma growth *in vivo*

**Figure 4:**
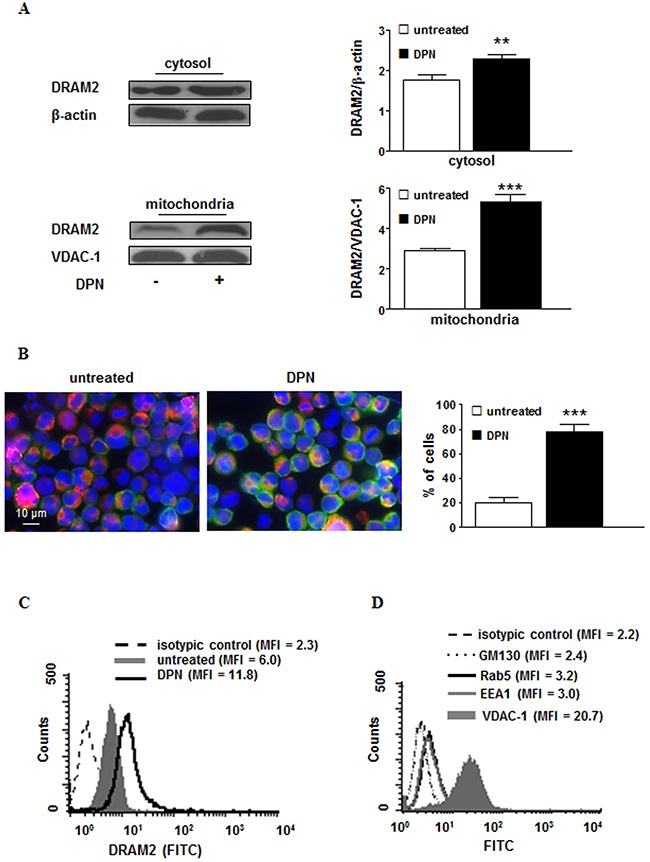
Cellular expression and localization of DRAM2 in HL cells after DPN treatment **A**. Western blot analysis of DRAM2 levels in cytosolic and mitochondrial fractions obtained from L-428 cells treated or not with 10 nM DPN for 24 hours. Blots shown are representative of 3 independent experiments (left). Densitometry analysis of DRAM2 levels is also shown. Values are expressed as means ± SD. **, *p* < 0.01; ***, *p* < 0.001 *versus* untreated cells (right). **B**. Immunofluorescence analysis of DRAM2 (green staining) distribution in mitochondria (red staining) in untreated (left) or 10 nM DPN-treated (right) L-428 cells for 24 hours. Cells were stained with Hoechst dye to reveal nuclei (blue staining) and observed by a scanning confocal laser microscope, with an objective of 60× with numeric aperture of 1.4. The images were acquired by the specific software IAS 2000. The percentages of cells in which DRAM2 localized at mitochondrial level are also shown and expressed as mean ± SD of the results obtained from 3 independent experiments. **C**. Representative flow cytometry analysis of purified mitochondria from L-428 cells treated or not with 10 nM DPN for 24 hours after staining with anti-DRAM2 monoclonal antibody. Broken line, isotype control staining; grey solid curve, DRAM2-labeled mitochondria from untreated cells, and black line, DRAM2-labeled mitochondria from 10 nM DPN-treated cells. **D**. Representative flow cytometry analysis of mitochondria preparation after staining with monoclonal antibodies to GM130 (dotted line), Rab5 (black line), EEA1 (grey line) or VDAC-1 (solid grey curve), to test its degree of purity. The isotype control is represented by the broken line. Results from one representative experiment out of 5 are shown. MFI, mean fluorescence intensity.

We next evaluated the *in vivo* anti-tumor activity of the ERβ agonist DPN in NOD/SCID mice xenografted with L-428 and L-540 cell lines. Mice received a 15 day treatment with DPN (3, 6, and 12 mg/kg, 5 days per week for 3 weeks) or vehicle control. No significant changes in weight or other signs of potential toxicity were observed during DPN treatment (data not shown). DPN significantly affected in a dose-dependent manner the *in vivo* growth of L-428 (Figure 3A) and L-540 (Supplementary Figure 4A), [tumor growth inhibition (TGI) = 58% and 52% at 12 mg/kg, respectively]. These findings were paralleled by a strong decrease in Ki-67 expression in L-428 (Figure 3B) and L-540 (Supplementary Figure 4B) cells, suggesting that DPN inhibited tumor cell proliferation. We also performed immunohistochemistry for LC3 and SQSTM1 expression in L-428 (Figure 3C) and L-540 (Supplementary Figure 4C) cells from mice treated with DPN or vehicle control. We detected a significant increase in the expression of LC3 (Figure 3C, enlarged micrograph shows localized distinct puncta typical of LC3-II appearance in autophagic cells). In contrast, DPN markedly decreased SQSTM1 expression in all HL tumors (L-428, Figure 3C; L-540, Supplementary Figure 4C). Additionally, these findings were not associated with an increase of tumor cell apoptosis and necrosis (Figure 3D and Figure 3E; Supplementary Figure 4D and Figure 4E).

### ERβ selective agonist DPN-induced damage-regulated autophagy modulator (DRAM)2 mediates autophagy in human HL cells

To identify the mechanisms underlying the ERβ activation-induced effects described above, genes potentially involved in autophagy induction were evaluated by RT^2^ Profiler Autophagy PCR array system in L-428 cells treated or not with 10 nM DPN. Differences in gene expression of ≥ 1.5-fold for DPN-treated cells *versus* untreated cells were considered (Table 1). Four genes, coding for the following proteins, were up-regulated: i) ULK1 protein kinase, a key regulator of autophagy initiation and progression [19]; ii) autophagy-related protein 9A (ATG9A), an essential member of autophagosome formation [20]; iii) the oncosuppressor p53 that, as a nuclear transcription factor, induces autophagy [21]; iv) DRAM2, a homologue of DRAM, whose role in autophagy modulation is still debated [22, 23]. To better define the potential mechanism of ERβ-dependent DRAM2 involvement in autophagy modulation, we analyzed by western blot the protein expression level of DRAM2 in L-428 cells after DPN treatment. We found significantly higher levels of DRAM2 protein in DPN-treated than in untreated L-428 cells at cytosolic (*p* < 0.01) and at mitochondrial (*p* < 0.001) levels (Figure 4A). DRAM2 re-localization at mitochondrial level from untreated or DPN-treated L-428 cells was also confirmed by immunofluorescence analysis (Figure 4B) and flow cytometry on purified mitochondria (Figure 4C). The purity of the mitochondrial preparation was tested by flow cytometry (Figure 4D).

**Table 1:** Changes in the expression of autophagy-related genes in DPN-treated L-428 HL cells compared with untreated cells

GeneBank	Symbol	Description	Treated *versus* untreated
NM_003565	ULK1	Unc-51-like kinase 1 (*C. elegans*)	1.50
NM_000546	TP53	Tumor protein p53	1.96
NM_178454	DRAM2	DNA-damage regulated autophagy modulator 2	1.51
NM_024085	ATG9A	ATG9 autophagy related 9 homolog A (*S. cerevisiae*)	2.96

**Figure 5:**
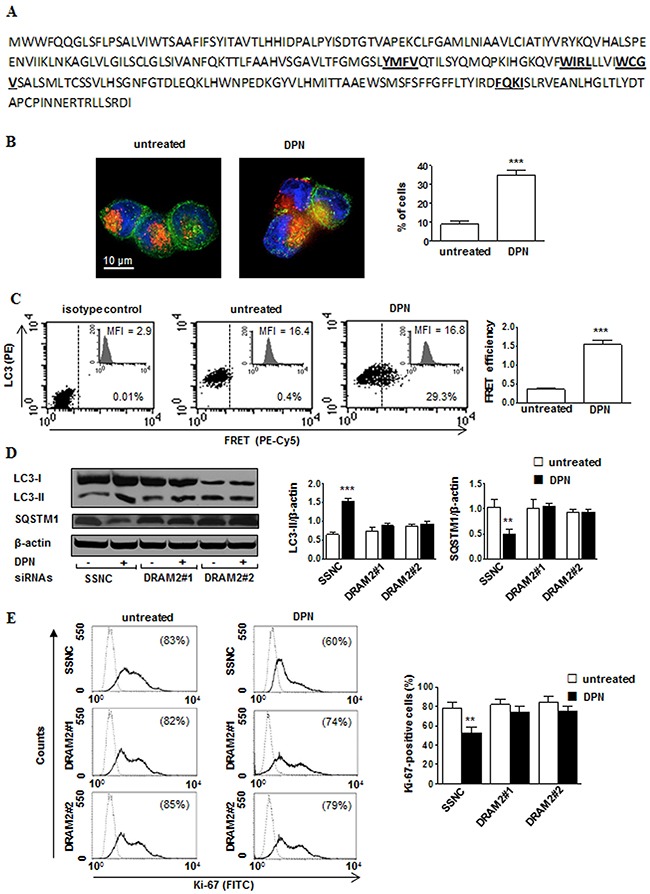
DPN-mediated induction of autophagy and inhibition of proliferation is mediated by DRAM2 in HL cells **A**. DRAM2 amino acid sequence. W/F/Y-x-x-L/I/V consensus sequence for the core LIR motif are underlined and bold. **B**. Immunofluorescence analysis of LC3 (green staining) and DRAM2 (red staining) from untreated (left) or 10 nM DPN treated (right) L-428 cells for 24 hours. Cells were counterstained with Hoechst dye to reveal nuclei (blue staining) and observed by a scanning confocal laser microscope with an objective of 60× with numeric aperture of 1.4. The images were acquired by the specific software IAS 2000. The percentages of cells in which LC3/DRAM2 co-localized occurres are shown and expressed as mean ± SD of the results obtained from 3 independent experiments. **C**. Representativeflow cytometry analysis of DRAM2 - LC3 association by FRET technique in untreated (middle) and 10 nM DPN treated (right) cells for 24 hours. DRAM2 protein was detected in FL4 channel (Cy5), LC3 in FL2 channel (PE), and FRET in FL3 channel (PE-Cy5). Isotype control is shown in left panel. Numbers indicate the percentage of FL3-positive events. Mean fluorescence intensity (MFI) is referred to DRAM2 expression. Bar graph shows the evaluation of FRET efficiency (FE), according to the Riemann algorithm of DRAM2-LC3 association. Results represent the mean ± SD from 3 independent experiments. ***, *p* < 0.001 *versus* untreated cells. **D**. Western blot analysis of LC3-II and SQSTM1 levels in cell lysates from L-428 cells transfected with Silencer Select Negative Control (SSNC) or DRAM2 siRNAs (DRAM2#1 and DRAM2#2) treated or not with 10 nM DPN for 24 hours. Blots shown are representative of 3 independent experiments (left). Densitometry analysis of LC3-II and SQSTM1 levels relative to β-actin is also shown (right). Values are expressed as means ± SD. **, *p* < 0.01; ***, *p* < 0.001 *versus* untreated cells. **E**. Cell proliferation was evaluated by flow cytometry measuring Ki-67 nuclear antigen expression in L-428 cells transfected with SSNC or DRAM2 siRNA (DRAM2#1 and DRAM2#2) treated or not with 10 nM DPN for 48 hours. Results from one representative experiment out of 3 are shown (left). Data are also reported as mean ± SD (right), **, *p* < 0.01 *versus* untreated cells.

Hereafter, we checked whether DRAM2 could bind LC3 so as to enhance autophagosome formation. The known consensus sequence for the core LC3-interacting region (LIR) motif is W/F/Y-x-x-L/I/V [24]. Therefore, we examined *in silico* DRAM2 sequence and we found that DRAM2 possesses four putative LIR motifs (Figure 5A). Accordingly, immunofluorescence analysis revealed a co-localization of LC3 with DRAM2 in a significant (*p* < 0.001) percentage of DPN-treated cells, as clearly demonstrated by yellow staining in micrograph (Figure 5B). We thus investigated the possible physical interaction of DRAM2 with LC3 by quantitative fluorescence resonance energy transfer (FRET) methodology. FRET efficiency clearly showed a negligible molecular association between DRAM2 and LC3 in untreated cells and a highly significant molecular interaction in DPN-treated cells (Figure 5C, *p* < 0.001), suggesting that DRAM2 - LC3 binding plays a crucial role in ERβ-mediated autophagy induction in HL cells. To confirm these data we silenced DRAM2 by transfection of L-428 and L-540 cells with DRAM2 siRNAs. Twenty-four hours after siRNA addition, DRAM2 expression decreased by about 50% as assessed by western blot (Supplementary Figure 5A and Figure 5B for L-428 and L-540 cells, respectively). Notably, we found that DRAM2 silencing was able to partially impair i) DPN-dependent induction of autophagy (i.e., increase of LC3-II levels and decrease of SQSTM1 levels; L-428, Figure 5D; L-540, Supplementary Figure 5C) and ii) DPN-dependent inhibition of proliferation (L428, Figure 5E; L-540, Supplementary Figure 5D).

## DISCUSSION

In this study we established, for the first time, a direct link between the anticancer activity of ERβ and autophagy induction in different HL cell lines. In particular, we found that the activation of ERβ with a specific agonist was capable of affecting HL tumor growth triggering a complex framework of events able to potently induce autophagy and impair cell cycle progression and cell proliferation. Dissecting the mechanisms underlying this activity, we found that a key actor in the ERβ activated pathway in these cells is represented by the autophagy-related molecule called DRAM2.

The role and the activity of ERβ signaling pathway in cancer cells is still poorly understood. A key role for ERβ in the inhibition of tumor growth in different forms of cancers has been hypothesized [10, 12, 25, 26]. In particular, a recent study carried out in breast cancer suggested that ERβ activation could inhibit cancer cell proliferation through a mechanism that bring into play a marked reduction of G2/M phase as well as triggering autophagy [14]. Interestingly, also in a male tumor such as seminoma, a testicular germ cell tumor, ERβ ligation was capable of impairing tumor growth enhancing the expression of autophagy-related markers [13]. In this context, ERβ-mediated autophagy could exert negative effects on cell growth through a targeted elimination of growth-promoting molecules, complexes and organelles so that proliferation results impaired [27]. Hence, autophagy and cell proliferation could represent mutually exclusive cell fates [27]. In line with this hypothesis, we found that induction of autophagy by specific ligation of ERβ by DPN was associated with a reduction of cancer cell proliferation both in cultured HL cells and, even more potently, in HL grafted in SCID mice (up to 60%).

Hence, it should be underscored that *per se* (i.e., given alone) an agonist of ERβ can significantly impair HL growth. Accordingly, the relevance of the induction of autophagy in order to hinder cancer cell proliferation gained the attention of a number of research groups in the recent years. For example, the possibility to induce autophagy with drugs able to target this pathway, such as rapamycin derivatives, has recently been considered extensively in basic and clinical studies [28].

When HL cells treated with DPN were analyzed for upregulation of autophagy-associated genes, using a PCR assay with a panel of 84 autophagy-related genes, only four genes were significantly upregulated (i.e., TP53, ULK1, DRAM2 and ATG9). This result suggests that ERβ-mediated autophagy induction is not determined by significant gene expression modification but it possibly involves post-translational events. The role of TP53, ULK1 and ATG9 in autophagy process has been well defined whereas that of DRAM2 has been less investigated [23]. Hence, we decided to study the role of DRAM2 in this context. We found for the first time that ERβ activation induced in HL cells i) an over-expression and re-distribution of DRAM2 in mitochondria, and ii) a molecular interaction of DRAM2 with LC3, the key molecule of autophagosome formation [29]. The experiments carried out to assess the localization of DRAM2 in cells after ERβ challenging clearly indicated that mitochondria could participate in the ERβ-mediated pathway leading to autophagy. This is in line with some literature data suggesting that DRAM2 could contribute to the peculiar form of autophagy leading to the engulfment of mitochondria in autophagic vacuoles called mitophagy [30]. In addition, the possibility that mitochondria-associated membranes (MAMs), that play a crucial role in autophagosome formation [31] being the interaction sites between mitochondria and certain subdomains of the endoplasmic reticulum [32], could participate in DRAM2-mediated autophagy cannot be ruled out. Moreover, our experiments with cells knocked down for DRAM2 gene expression suggested that DRAM2 was a prerequisite for ERβ-induced induction of autophagy and inhibition of proliferation.

On these bases, and taking into account the availability in the market of several ERβ agonists, we can hypothesize that future research should be aimed at evaluating the potential therapeutic effectiveness of selective ERβ agonists, including natural phytoestrogens or food supplements, in specific combinatory clinical trials with conventional chemotherapeutic agents, aimed at assessing the anti-tumor effects of these agents.

## MATERIALS AND METHODS

### Cell lines and treatments

The HL cell lines L-428, KM-H2, L-540 and HDLM-2 were purchased from the German Collection of Microorganisms and Cell Cultures (DSMZ, Braunschweig, Germany). The identity of the cell lines was authenticated by multiplex PCR of minisatellite markers that revealed a unique DNA profile. Cell cultures were also tested for the presence of Mycoplasma (Mycoplasma Detection Kit, Invivogen, San Diego, CA, USA) before the initiation of this study. Cells were cultured in RPMI-1640 medium (Gibco BRL, Grand Island, NY, USA) containing 10% fetal bovine serum (FBS; Euroclone, Euroclone, Pero, Milan, Italy), 2 mM glutamine (Sigma, St. Louis, MO, USA), and 50 ug/mL gentamycin (Sigma) at 37 °C in a humidified 5% CO_2_ atmosphere. For estrogen-free cell culture, cells were cultured in phenol red-free RPMI-1640 containing 10% dextran-coated charcoal-stripped FBS (Euroclone) for 3 days before specific treatments. Cells were treated with the ERβ agonist DPN (10 nM; Sigma) and in selective experiments a pre-treatment with 1 mM 3-MA (Sigma) or 1 μM HCQ (Sigma) was carried out. Where indicated cells were also treated in the presence of lysosomal inhibitors E64d and PepA (both at 10 μg/ml; Sigma) for the last 2 hours of culture. Control treatments with the vehicle in which tested compounds were solubilized (ethanol or dimethyl sulfoxide) were included. All experiments were performed at least three times. Total incubation time was 24-48 hours (depending on experiments).

### Gene silencing by siRNA

HL cells were transfected using Lipofectamine® RNAiMAX (Invitrogen, Carlsbad, CA, USA) with the following Silencer Select siRNAs: for *ESR2* (ERβ), i) ERβ#1, sense, GAUUAUAUUUGUCCAGCUATT, antisense, UAGCUGGACAAAUAUAAUCAT (#S4828, Ambion, Inc., Austin, TX, USA) and ii) ERβ#2, sense, AGUGUACAAUCGAUAAAAATT, antisense, UUUU UAUCGAUUGUACACUGA (#S4826, Ambion); for *DRAM2*, i) DRAM2#1, sense, GGCUUUAC CUUAUAUCAGUTT, antisense, ACUGAUAUAAG GUAAAGCCGG (#S43283, Ambion) and ii) DRAM2#2, sense, GGUUAUCUGGUGUGGAGUATT, antisense, UACUCCACACCAGAUAACCAA (#S43282, Ambion). Silencer Select Negative Control siRNA (SSNC, #4390846, Ambion) was also used as negative control according to the manufacturer's instructions. Twenty-four hours after siRNA transfection, cells were treated with 10 nM DPN for 24 hours.

### Flow cytometry analysis

#### Apoptosis, proliferation and cell cycle

Apoptosis was quantified using a fluorescein isothiocyanate (FITC)-conjugated AV and PI detection kit according to the manufacturer's protocol (Marine Biological Laboratory, Woods Hole, MA, USA). This assay enables identification of both early (AV positive/PI negative) and late apoptotic or necrotic (PI positive) cells.

Proliferation was evaluated by measuring Ki-67 nuclear antigen expression using the phycoerythrin (PE)-mouse anti-human Ki-67 Set according to the manufacturer's protocol (BD Biosciences, San Jose, CA, USA). To analyze cell cycle progression, cells were synchronized at G1/S boundary by treatment with 0.7 ug/ml aphidicolin (Sigma), a specific DNA polymerase inhibitor, for 18 hours. After this time, cells were washed and then treated with 3-MA and/or DPN, as stated above, or left untreated. Cells were then analyzed for cell cycle by the 5-bromo-2-deoxy-uridine (BrdU)/anti-BrdU method. Briefly, cells were pulse-labeled for 45 minutes with 30 μM of BrdU (Sigma). After this time, cells were processed as previously reported and then analyzed for cell cycle by a biparametric flow cytometry analysis [33]. Samples were analyzed by collecting FL2 red fluorescence in a linear scale at 620 nm and FL1 green fluorescence in logarithmic scale at 512 nm.

### *In Vitro* colony formation assay

The suspended L-428 cells (number = 2,500)were plated in culture dishes with 1 ml of semisolid methylcellulose culture medium (Methocult H4034; Stem Cell Technologies, Meylan, France) in the presence or absence of DPN 10 nM. Duplicates aliquots (1 mL) were plated in 35-mm Petri plates and incubated further at 37°C. Colonies were counted at 10 days.

### Quantitative fluorescence resonance energy transfer (FRET) analysis

We applied quantitative FRET analysis in order to study cell-by-cell the molecular association of LC3 with DRAM2 in different experimental conditions in entire cells. As previously reported [34], cells were fixed, permeabilized, and then labeled with antibodies tagged with donor (PE) or acceptor (Cyanine 5, Cy5) dyes. Cell staining was performed using rabbit anti-LC3 polyclonal antibody (Abgent, San Diego, CA, USA) and mouse anti-DRAM2 monoclonal antibody (Millipore, Billerica, MA, USA), followed by Cy5-labeled anti-rabbit (Abcam, Cambridge, UK) and PE-labeled anti-mouse (BD Biosciences) antibodies. DRAM2 protein was detected in FL4 channel (Cy5), LC3 in FL2 channel (PE), and FRET in FL3 channel (PE-Cy5). For the determination of FRET efficiency, changes in fluorescence intensities of donor-plus-acceptor-labelled cells were compared to the emission signal from cells labelled with donor- and acceptor-only fluorophores. As a further control, the cross-reactivity among all the different primary and secondary antibodies was also assessed. All data were corrected for background by subtracting the binding of the isotype controls. Efficient energy transfer resulted in an increased acceptor emission on cells stained with both donor and acceptor dyes. Statistical analysis was performed by pooling together data obtained from three independent measurements and calculating the FRET efficiency (FE) according to Riemann [35] by using the following algorithm:

FE = [FL3DA – FL2DA/a – FL4DA/b]/FL3DA

in which A is the acceptor and D the donor and where a = FL2D/FL3D and b = FL4A/FL3A.

For flow cytometer experiments, acquisition was performed on a FACSCalibur flow cytometer (BD Biosciences) equipped with a 488 argon laser and with a 635 red diode laser and at least 30,000 events per sample were run. Data were analyzed using the Cell Quest Pro software (BD Biosciences).

### Isolation of mitochondria for flow cytometry analysis

Cells were suspended in Homo-buffer (10 mM Hepes, pH 7.4; 1 mM ethylene glycol-bis(-aminoethyl ether) N,N’,N”-tetraacetic acid, 0.1 M sucrose, 5% bovine serum albumin, 1 mM phenylmethylsulfonyl fluoride and complete protease inhibitor cocktail (Roche Diagnostics GmbH, Mannheim, Germany), and maintained for 10 minutes on ice. After this time, cells were homogenized with about 100 strokes of a Teflon homogenizer with B-type pestle as previously reported [36] for 10 minutes at 4°C to remove intact cells and nuclei and the supernatants were further centrifuged at 10.000 x g at 4°C for 10 minutes to precipitate the heavy membrane fractions (enriched in mitochondria). These fractions were then purified by standard differential centrifugation. Protein content in the mitochondrial preparation was determined by a spectrophotometric method using bovine serum albumin as standard. We tested the purity of the mitochondrial preparation by flow cytometry after staining with monoclonal antibodies specific to endolysosomal compartment markers EEA1 (Santa Cruz Biotechnology, Santa Cruz, CA, USA), Rab5 (Santa Cruz), and Golgi vesicle antigen GM130 (Santa Cruz), followed by incubation with Alexa Fluor 488-anti-mouse antibody (Thermo Fisher Scientific, Waltham, MA, USA). As negative control we used mouse IgG1 immunoglobulin (Sigma) and as positive control monoclonal antibody to Voltage-dependent anion-selective channel protein 1 (VDAC-1, Santa Cruz).

### Preparation of mitochondria and cytosol fractions for western blot analysis

Mitochondria and cytosol fraction from HL cells were obtained using the Mitochondria Isolation Kit for Cultured Cells (Thermo Fisher Scientific) following manufacturer's instruction. Protein content was determined by a spectrophotometric method using bovine serum albumin as standard.

### SDS-PAGE and western blot

Cells were lysed in RIPA buffer [100 mM tris(hydroxymethyl)aminomethane (Tris)-HCl pH 8, 150 mM NaCl, 1% Triton X-100, 1 mM MgCl_2_] in the presence of a complete protease-inhibitor mixture (Roche Diagnostics GmbH). Protein content was determined by the Bradford assay (Bio-Rad Laboratories, Richmond, CA, USA). Samples were loaded onto SDS-PAGE and, after electrophoresis, proteins were transferred onto nitrocellulose membrane (GE Healthcare, Pittsburgh, PA, USA) by means of a Trans-Blot transfer cell (Bio-Rad Laboratories). The membranes were then blocked in 5% nonfat milk and incubated with the appropriate antibodies in Tris-buffered saline (TBS) containing 0.1% Tween 20 and 5% nonfat milk. Mouse anti-human LC3B (Novus Biologicals, Littleton, CO, USA), rabbit anti-human SQSTM1 (Sigma), mouse anti-human ERβ (clone 1531, Santa Cruz), and mouse anti-human DRAM2 (Millipore) were used as primary antibodies. Peroxidase-conjugated goat anti-mouse IgG (Bio-Rad Laboratories) or anti-rabbit IgG (Bio-Rad Laboratories) were used as secondary antibodies and the reactions were developed using the ECL Prime Western Blotting Detection Reagent (GE Healthcare). To ensure the presence of equal amounts of proteins, the membranes were reprobed with rabbit anti-human β-actin (Sigma), rabbit anti-human glyceraldehyde-3-phosphate dehydrogenase (GAPDH, Sigma), or mouse anti-human VDAC-1 (Santa Cruz) antibodies. Quantification of protein expression was performed by densitometry analysis of the autoradiograms (GS-700 Imaging Densitometer, Bio-Rad Laboratories).

### RNA isolation and quantitative real-time PCR

Total RNA (1 μg) extracted from HL cells (RNeasy Mini Kit, Qiagen, Valencia, CA, USA) was retrotranscribed in cDNA using an RT^2^ First Strand kit according to the manufacturer's protocol (SABioscience, Frederick, MD, USA). To perform gene expression profiling, we used SYBR Green based-technology and the RT^2^ Profiler™ PCR Array Human Autophagy (SABioscience) that analyzes the expression of 84 genes involved in autophagy. The RT^2^ Profiler™ PCR Array Human Autophagy includes specific validated primer sets and PCR master mixes (SABioscience). Real-time PCRs were performed in 96-well plate format using the ABI 7000 Real-Time PCR System (Applied Biosystems, Foster City, CA, USA). The parameters for PCR amplification were 50°C for 2 minutes, 95°C for 10 minutes followed by 40 cycles of 95°C for 15 seconds, and 60°C for 1 minute. Fold changes of autophagic gene expression in experimental samples relative to the control samples were calculated using the ΔCt method.2 The ΔCt value of each sample was normalized by up to a total of 5 housekeeping genes (*β2-microglobulin*, *hypoxanthine phosphoribosyltransferase 1*, *ribosomal protein L13a*, *GAPDH*, and *β-actin*).

### Immunofluorescence

Cells, fixed with 4% paraformaldehyde and permeabilized by 0.5% (v/v) Triton X-100, were incubated with the following antibodies: rabbit anti-LC3 polyclonal antibody (Abgent), mouse anti-DRAM2 monoclonal antibody (Millipore), mouse anti-mitochondria monoclonal antibody (Millipore) as primary antibodies and AlexaFluor 488 anti-rabbit IgG (Thermo Fisher Scientific) or AlexaFluor 594 anti-mouse IgG (Thermo Fisher Scientific) as secondary antibodies. Finally, samples were counterstained with Hoechst 33258 (Sigma) and then mounted in glycerol/PBS (ratio 1:1, pH 7.4).

Samples were observed and acquired through a scanning confocal laser microscope (Leica Microsystems). For morphometric analyses at least 100 cells were evaluated for each experimental point by analyzing merge images acquired by software package IAS 2000 (Delta Sistemi, Rome, Italy).

### Transmission electron microscopy (TEM) studies

For TEM examination, HL cells were fixed in 2.5% cacodylate-buffered (0.1 M, pH 7.2) glutaraldehyde (TAAB, Aldermaston, UK) for 20 minutes at room temperature and post-fixed in 1% OsO4 (Electron Microscopy Sciences, Hatfield, PA, USA) in cacodylate buffer for 1 hour at room temperature. Fixed specimens were dehydrated through a graded series of ethanol solutions and embedded in Agar 100 (Electron Microscopy Sciences). Ultrathin sections were collected on 200-mesh grids and counterstained with uranyl acetate (Electron Microscopy Sciences) and lead citrate. Sections were observed with a Philips 208 electron microscope at 80 kV.

### Activity of DPN in tumor-bearing nonobese diabetic/severe combined immunodeficient (NOD/SCID) mice

Six- to eight-week-old NOD/SCID mice (20 to 25 g) were purchased from Charles River (Milan, Italy) and xenografted with L-428 (20 × 10^6^ cells/mouse) or L-540 (25 × 10^6^ cells/mouse) cells by subcutaneously inoculation into the left flank. When the tumor volume reached approximately 100 mm^2^, the mice were randomly assigned to receive either short- or long-term treatment with DPN by intraperitoneal injection [working solution containing ≤ 10% dimethyl sulfoxide (DMSO) (v/v)] or vehicle [≤ 10% DMSO solution (v/v)]. As previously reported [37], mice treatment with a 10% DMSO solution failed to affect tumor cell signaling likely due to *in vivo* dilution of DMSO as well as its rapid metabolic clearance. Short-term treatment, which consisted of DPN (12 mg/kg/5 day, intraperitoneally), was used to assess the apoptotic/necrotic areas, tumor proliferation, and autophagy. Experiments were performed on at least two separate occasions using three mice per treatment group. For long-term treatment, mice were treated with DPN (3, 6, 12 mg/kg/5 day/3 weeks, intraperitoneally). The tumor volumes were calculated using the following formula: (a × b^2^) / 2, where a and b represent the longest and shortest diameters, respectively. TGI was defined as (1 – [T/C] × 100), where T and C represent the mean tumor volumes in the treated and vehicle-treated controls, respectively. Experiments were performed on at least two separate occasions using five mice per treatment group. Animal experiments were performed according to EU 86/109 Directive (D.L. 116/92 and following additions) and approved by the institutional Ethical Committee for Animal Experimentation of the Humanitas Clinical and Research Center.

### Histological analysis and immunohistochemistry

Sections (2-µm thick) from formalin-fixed, paraffin-embedded tumor nodules were stained with hematoxylin and eosin or with anti-human Ki-67 (Dako, Cernusco sul Naviglio, Milan, Italy), anti-LC3 (Abgent), and anti-SQSTM1 (Sigma) specific antibodies. Images were analyzed using Image-Pro Analyzer software. Tumor apoptosis and necrosis were detected by TUNEL staining (Roche Diagnostics GmbH). The sections were examined under a light microscope (IX51; Olympus). Image analysis was performed using the open source software ImageJ [38].

### Statistical analysis

Statistical analysis was performed with the statistical package Prism 6 (GraphPad Software). *In vitro* statistical analysis was performed by Student's t test. To test the probability of significant differences between the vehicle and DPN-treated tumor nodules, two-way analysis of variance (ANOVA) was used, and individual group comparisons were evaluated using Bonferroni's test. *p* values of < 0.05 were considered significant.

## SUPPLEMENTARY MATERIALS FIGURES


