# Supplementary material for: Estrogen receptor β ligation inhibits Hodgkin lymphoma growth by inducing autophagy
Source: Oncotarget. 2016 Dec 28;8(5):8522–35. doi: 10.18632/oncotarget.14338 (PMC5352419; doi:10.18632/oncotarget.14338)
Supplement: Supplementary file 1 [file oncotarget-08-8522-s001.pdf]

Estrogen receptor β ligation inhibits Hodgkin lymphoma growth by inducing autophagy

SUPPLEMENTARY FIGURES

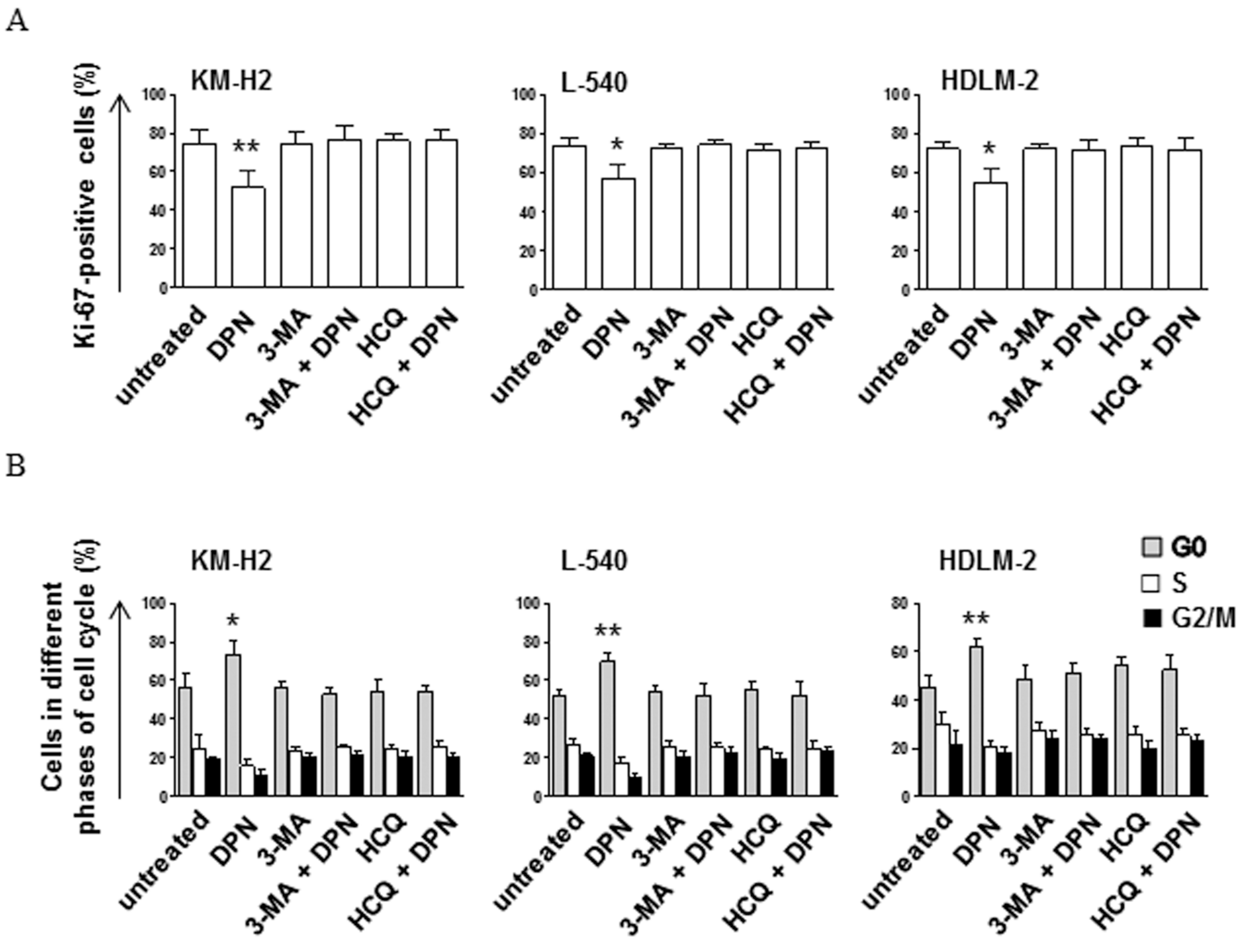

Supplementary Figure S1: DPN reduces cell proliferation and alters cell cycle progression in KM-H2, L-540, and HDLM-2 cell lines. A. Cell proliferation was evaluated by flow cytometry measuring Ki-67 nuclear antigen expression in KM-H2, L-540, and HDLM-2 cells treated or not with 10 nM DPN for 48 hours in the presence or absence of 3-MA or HCQ. For each cell line, data are reported as mean ± SD from 3 independent experiments. *, $p < 0.05$ and **, $p < 0.01$ *versus* untreated cells. **B.** Cell cycle progression was evaluated by flow cytometry using the BrdU/anti-BrdU method in synchronized KM-H2, L-540, and HDLM-2 cells treated or not with 10 nM DPN for 48 hours in the presence or absence of 3-MA or HCQ. For each cell line, data are reported as mean ± SD from 3 independent experiments. *, $p < 0.05$ and **, $p < 0.01$ *versus* untreated cells.

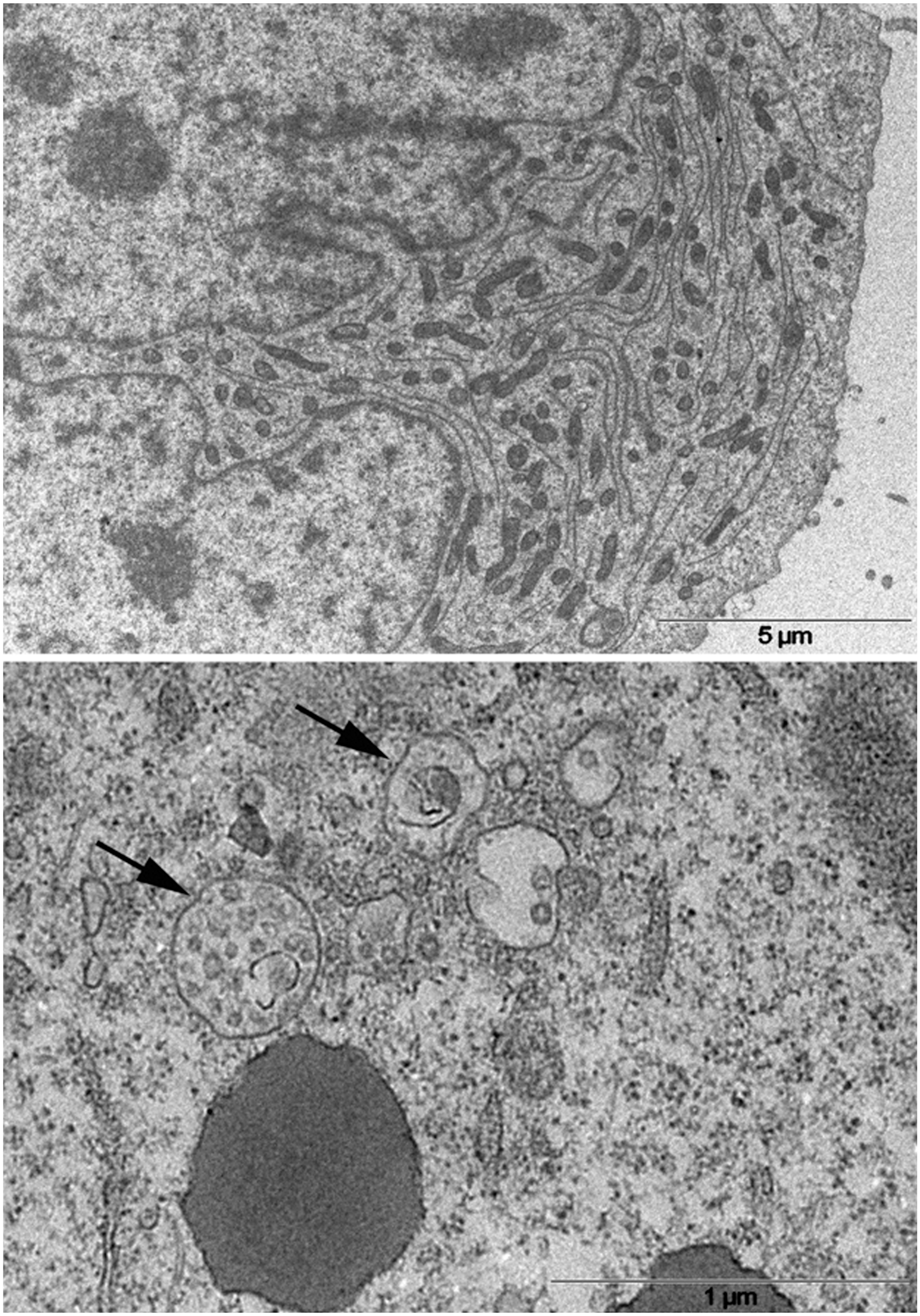

Supplementary Figure S2: DPN induces autophagy in L-540 cell line. TEM analysis performed on L-540 cells untreated (top panel) or treated (bottom panel) with 10 nM DPN for 24 hours and observed by a Philips 208 electron microscope at 80 kV, equipped with Mega Wiew II, Olympus soft imaging solutions. Autophagic vacuoles are indicated (arrows). Results from one representative experiment out of 3 are shown.

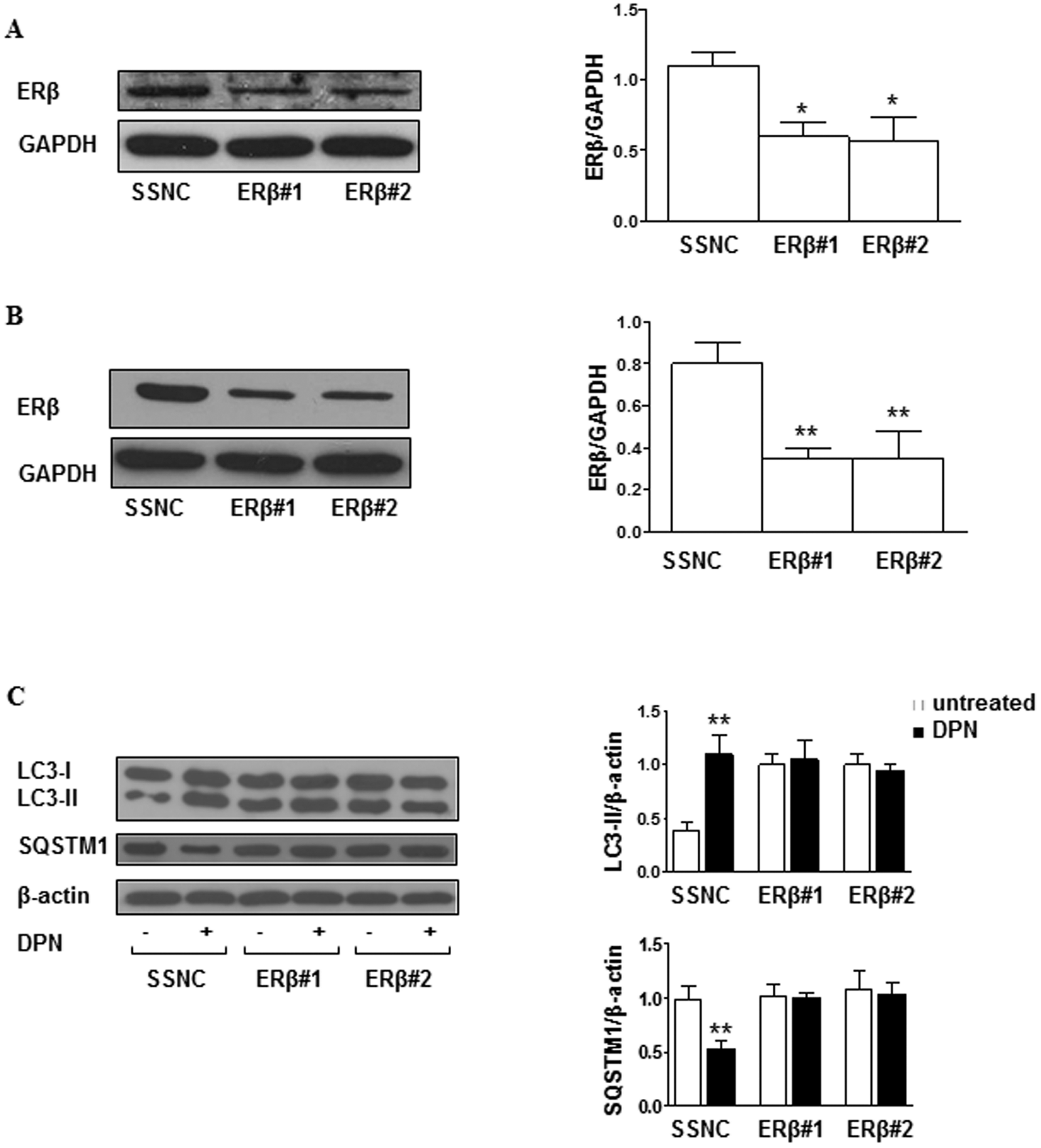

Supplementary Figure S3: ERβ siRNA inhibit DPN-induced autophagy. A,B. Western blot analysis of ERβ levels in cell lysates from L-428 (A) and L-540 (B) cells after 24 hours of Silencer Select Negative Control (SSNC) or siRNA (ERβ#1 and ERβ#2) addition. **C.** Western blot analysis of LC3-II and SQSTM1 levels in cell lysates from L-540 cells transfected with Silencer Select Negative Control (SSNC) or ERβ siRNA (ERβ#1 and ERβ#2) and treated or not with 10 nM DPN for 24 hours. Blots shown are representative of 3 independent experiments (left). Densitometry analysis of LC3-II levels relative to β-actin is also shown (right). Values are expressed as mean ± SD. **, $p \leq 0.01$ *versus* untreated cells.

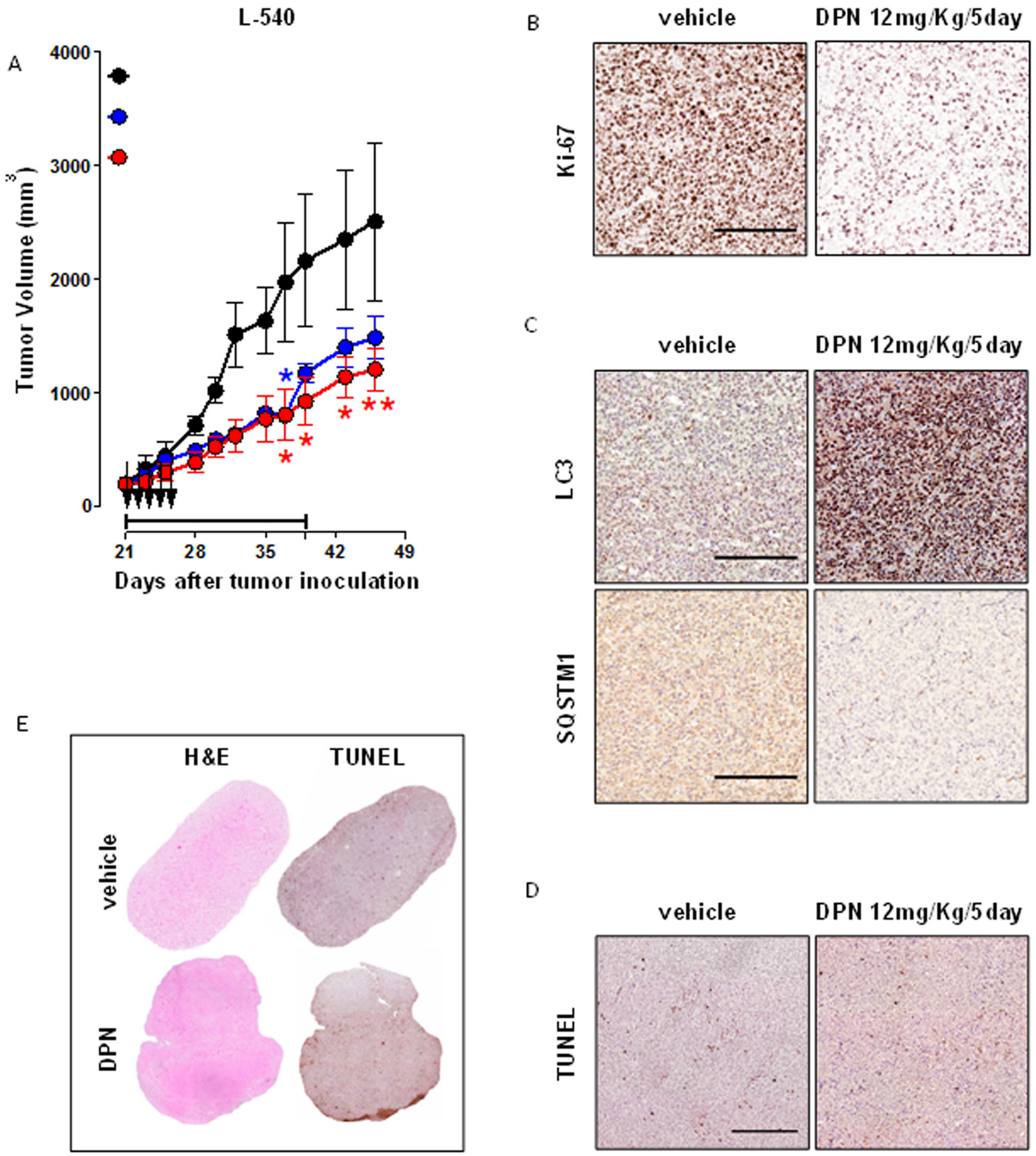

Supplementary Figure S4: DPN induces growth inhibition and autophagy in L-540 xenografts in NOD/SCID mice. **A.** NOD/SCID mice bearing 100-mm^3 L-540 tumor nodules were randomly assigned to receive 15-day treatment with DPN by intraperitoneal injection (3 mg/kg, 5 days/week for 3 weeks, blue; 6 mg/kg, 5 days/week for 3 weeks, green; 12 mg/kg, 5 days/week for 3 weeks, red) or vehicle control (black). Black arrows indicate DPN treatment administration whereas treatment duration is indicated by horizontal capped black lines (days 28-46). The mean (± SEM) tumor volumes are shown. *, $p < 0.05$ and **, $p < 0.01$ *versus* vehicle control. **B.** Ki-67 and **C.** LC3 and SQSTM1 staining of L-540 tumors treated with DPN (12 mg/kg/day, 5 days) or vehicle control. In the Ki-67-, LC3-, and SQSTM1-stained sections, brown staining represents a positive signal within the tumor. Objective lens, 0.75 numerical aperture (NA) dry objective; original magnification, 20x. Scale bar, 100 µm. **D.** In L-540 tumors treated with DPN (12 mg/kg/day, 5 days) or vehicle control, apoptotic tumor cells were detected by TUNEL staining and were revealed as brown nuclei using 3,3'-diaminobenzidine for light microscopy analysis. Objective lens, 0.08 NA dry objective; original magnification, 20x. Scale bar, 50 µm. **E.** Representative histological images of entire tumor sections from L-540 tumors treated with DPN (12 mg/kg/day, 5 days) or vehicle control. Tumor tissue morphology was detected via hematoxylin and eosin (H&E) staining. The tumor necrotic areas were detected via TUNEL staining and were visualized as brown. Objective lens, 0.08 NA dry objective; original magnification, 2x.

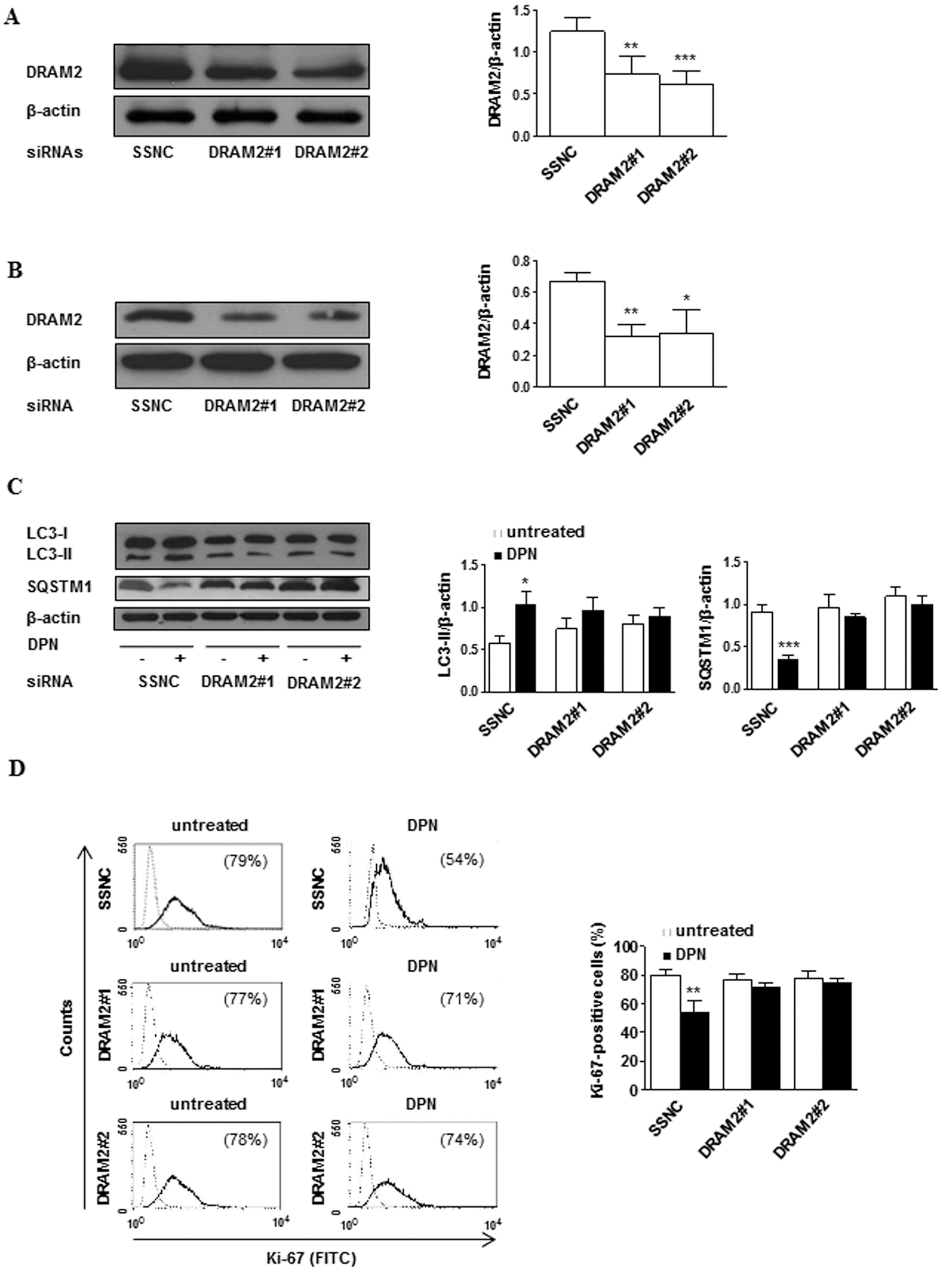

Supplementary Figure S5: DRAM2 siRNA impact on autophagy and proliferation in L-540 HL cells. A,B. Western blot analysis of DRAM2 levels in cell lysates from L-428 (A) and L-540 (B) HL cells transfected with Silencer Select Negative Control (SSNC) or DRAM2 siRNA (DRAM2#1 and DRAM2#2). Blots shown are representative of 3 independent experiments (left). Densitometry analysis of DRAM2 levels relative to β-actin is also shown (right). Values are expressed as mean ± SD. *, $p < 0.05$, **, $p < 0.01$ and ***, $p < 0.001$ *versus* SSNC transfected cells. **C.** Western blot analysis of LC3-II and SQSTM1 levels in cell lysates from L-540 cells transfected with Silencer Select Negative Control (SSNC) or DRAM2 siRNA (DRAM2#1 and DRAM2#2) treated or not with 10 nM DPN for 24 hours. Blots shown are representative of 3 independent experiments (left). Densitometry analysis of LC3-II (upper panel) and SQSTM1 (bottom panel) levels relative to β-actin is also shown (right). Values are expressed as mean ± SD. *, $p < 0.05$ and ***, $p < 0.001$ *versus* untreated cells. **D.** Cell proliferation was evaluated by flow cytometry measuring Ki-67 nuclear antigen expression in L-540 cells transfected with Silencer Select Negative Control (SSNC) or DRAM2 siRNA (DRAM2#1 and DRAM2#2), treated or not with 10 nM DPN for 48 hours. Data are reported as mean ± SD from 3 independent experiments. *, $p < 0.05$ *versus* untreated cells.
